# 

**Published:** 1860-08

**Authors:** 


VOLUME XIV.

### DENTAL REGISTER,

### OF THE WEST.

This volume will be issued in 6 months, commencing with the July Number, and closing with the December Number.

The price is ONE DOLLAR, for this volume.

**NOW IS THE TIME TO SUBSCRIBE.**

*Send on your Dollar and have your name enrolled.*

**TERMS - - STRICTLY CASH, IN ADVANCE.**

A FEW ADVERTISEMENTS WILL BE INSERTED AS FOLLOWS.

One Page, - - - - - $30.

Half Page, - - - - $20.

Quarter Page. - - - - $15.

